# Application of Functional Magnetic Resonance Imaging in the Diagnosis of Parkinson’s Disease: A Histogram Analysis

**DOI:** 10.3389/fnagi.2021.624731

**Published:** 2021-05-11

**Authors:** Dafa Shi, Haoran Zhang, Siyuan Wang, Guangsong Wang, Ke Ren

**Affiliations:** Department of Radiology, Xiang’an Hospital of Xia Men University, Xiamen, China

**Keywords:** Parkinson’s disease, functional MRI, amplitude of low-frequency fluctuation, histogram analysis, least absolute shrinkage and selection operator, machine learning

## Abstract

This study aimed to investigate the value of amplitude of low-frequency fluctuation (ALFF)-based histogram analysis in the diagnosis of Parkinson’s disease (PD) and to investigate the regions of the most important discriminative features and their contribution to classification discrimination. Patients with PD (*n* = 59) and healthy controls (HCs; *n* = 41) were identified and divided into a primary set (80 cases, including 48 patients with PD and 32 HCs) and a validation set (20 cases, including 11 patients with PD and nine HCs). The Automated Anatomical Labeling (AAL) 116 atlas was used to extract the histogram features of the regions of interest in the brain. Machine learning methods were used in the primary set for data dimensionality reduction, feature selection, model construction, and model performance evaluation. The model performance was further validated in the validation set. After feature data dimension reduction and feature selection, 23 of a total of 1,276 features were entered in the model. The brain regions of the selected features included the frontal, temporal, parietal, occipital, and limbic lobes, as well as the cerebellum and the thalamus. In the primary set, the area under the curve (AUC) of the model was 0.974, the sensitivity was 93.8%, the specificity was 90.6%, and the accuracy was 93.8%. In the validation set, the AUC, sensitivity, specificity, and accuracy were 0.980, 90.9%, 88.9%, and 90.0%, respectively. ALFF-based histogram analysis can be used to classify patients with PD and HCs and to effectively identify abnormal brain function regions in PD patients.

## Introduction

Parkinson’s disease (PD) is one of the most common clinically progressive neurodegenerative diseases worldwide, with prevalence second only to Alzheimer’s disease, and it affects more than 10 million people worldwide (Kim et al., 2017; Srivastav et al., 2017). Early diagnosis and treatment of PD are crucial to stop its progression in the initial stages (Chen et al., 2014; Adeli et al., 2016; Heim et al., 2017). In the early stage of PD, the main manifestations are non-motor symptoms, which are nonspecific and difficult to diagnose (Peng et al., 2017; Cigdem et al., 2018; Tuovinen et al., 2018; Rubbert et al., 2019). However, advancements in neuroimaging and machine learning technologies have led to an increasing role of such technologies in the accurate diagnosis of PD (Chen et al., 2014; Szewczyk-Krolikowski et al., 2014; Peng et al., 2017; Amoroso et al., 2018).

Resting-state functional magnetic resonance (rs-fMRI; Qin et al., 2019; Zhou Z. W. et al., 2019) is one of the most commonly used techniques for neuroimaging. The amplitude of low-frequency fluctuation (ALFF; Zhang et al., 2017; Xu et al., 2019), which can detect the amplitude of spontaneous brain fluctuations, is one of the most commonly used fMRI measurements.

Radiomics has been widely used in clinical oncology studies (Ji et al., 2020; Zhao K. et al., 2020); the medical images can be converted into feature sets that can be used to characterize tumor characteristics by a series of algorithms (Lambin et al., 2012; Ji et al., 2020). Radiomics has now been widely used in the study of neuropsychological diseases, their diagnosis, and their neurological mechanism (Sun et al., 2018; Huang K. et al., 2019; Mo et al., 2019; Wang et al., 2020). Histogram analysis is the most commonly used radiomic feature extraction method, which is widely used in neuroimaging research (Cui et al., 2016; Sun et al., 2018; Huang K. et al., 2019; Zhou et al., 2020). To our knowledge, there is no existing study that has used histogram analysis to diagnose PD.

Therefore, this study aimed to explore the value of using ALFF-based histogram analysis in the diagnosis of PD and to investigate the regions of the most important discriminative features and their contribution to classification discrimination in order to explore its potential pathological mechanism.

## Materials and Methods

### Subjects

The data in this article were obtained from a public database^1^ (Hu et al., 2015) including 41 healthy controls (HCs) and 59 PD patients. Previous studies (Varoquaux, 2018; Gorriz et al., 2021) have demonstrated that cross-validation in a small sample size leads to large error bars, and the predictive power of the fitted classifiers is arguable. Varoquaux (2018) also pointed out that the best resolution was to test the model performance across several datasets. Consequently, we divided the subjects into a primary set (32 HCs and 48 PDs) for training the model and a validation set (nine HCs and 11 PDs) for testing the model according to the order in which they entered the group based on an 8:2 ratio (to ensure a balanced ratio of PD and HC between the two groups). The clinical data obtained from each subject included age, sex, years of education, and Mini-Mental State Examination (MMSE) scores.

### Image Acquisition

High-resolution three-dimensional (3D) T1-weighted structure images and the rs-fMRI data of each participant were collected with a 3T Siemens MRI scanner (Siemens Healthineers, Erlangen, Germany). The parameters were as follows (Hu et al., 2015): 3D T1-weighted anatomical images: repetition time (TR)/echo time (TE) = 2,530/3.43 ms, field of view (FOV) = 256 × 256 mm, slice thickness = 1.3 mm, slice interval = 0.5 mm, slices = 128, matrix = 256 × 192, flip angle = 7°; rs-fMRI images: TR/TE = 2,000/30 ms, FOV = 220 × 220 mm, voxel size = 3.4 × 3.4 × 3.5 mm^3^, slice interval = 0.6 mm, slices = 31, matrix = 64 × 64, and flip angle = 90°.

### Data Preprocessing and ALFF Calculation

The data obtained in this study are the mean ALFF images. The data had already been preprocessed and the mean ALFF had been calculated. This processing is a standardized preprocessing step. The detailed mean ALFF preprocessing and calculation procedure can be found in a previous study (Hu et al., 2015). Briefly, the procedure includes the removal of the first 10 time points of the data, slice timing and realignment (subjects whose head motion parameters exceeded 2.5 mm of the maximum translation displacement or 2.5° of angular motion were excluded), image registration, spatial standardization, and image resampling (3 × 3 × 3 mm^3^), spatial smoothing using a 6 × 6 × 6-mm^3^ full-width half-maximum Gaussian kernel, high-pass filtering (0.01–0.08 Hz), and linear drift removal. The ALFF calculation and mean standardization were further performed on the preprocessed rs-fMRI data.

### Histogram Feature Extraction

**Figure 1:**
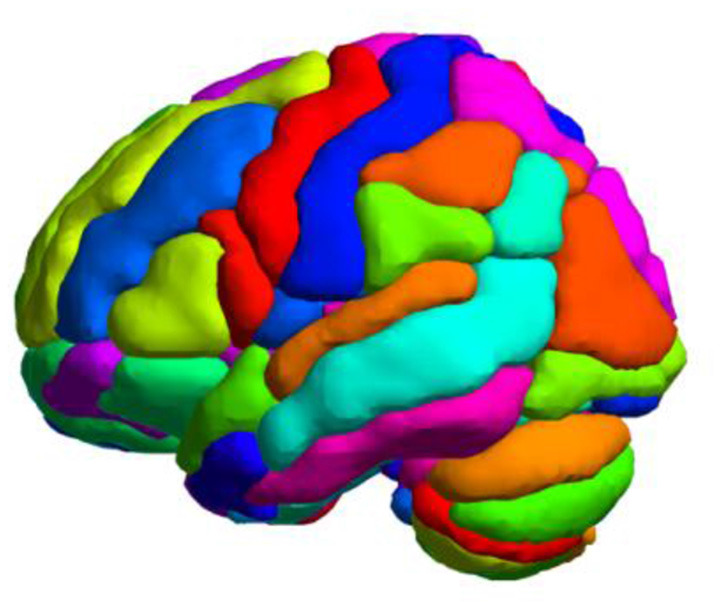
Whole-brain parcellation with the Automated Anatomical Labeling (AAL) 116 template.

Previous studies (Balagurunathan et al., 2014; Zhao et al., 2016; Berenguer et al., 2018) have demonstrated that histogram statistical features are reproducible and easy to interpret. Meanwhile, if the feature dimension is too high, it is easy for the model to fall into a “curse of dimensionality.” Consequently, in this study, we segmented the individual mean ALFF map into 116 regions of interest (ROIs) using the Automated Anatomical Labeling (AAL) 116 atlas (Figure 1), which consists of 90 subregions in the cerebrum and 26 subregions in the cerebellum. We extracted 11 intensity-based histogram features of the ALFF in each ROI, including the mean, minimum, maximum, range, standard deviation, variance, median, skewness, kurtosis, 10th percentile, and 90th percentile. The definitions and details of the features are described elsewhere (Aerts et al., 2014; Sun et al., 2018; Zhao K. et al., 2020). We extracted a total of 1,276 features for each subject.

### Feature Selection, Model Construction, and Evaluation

First, *Z*-normalization was performed before feature selection. In the primary set, a two-sample *t*-test was performed on the 1,276 histogram features, and the features with *P* < 0.05 were selected for the next analysis. If the correlation coefficient of the two variables was greater than 0.9, we deleted the latter of the two variables to reduce the multicollinearity between the variables (Tang et al., 2019). For the selected features, the least absolute shrinkage and selection operator (LASSO) logistic regression based on 10-fold cross-validation was used for further feature selection and data dimensionality reduction in the primary set, which used the mean squared error (MSE) and the minimum *λ* as the feature selection criteria. The LASSO logistic regression is suitable for the regression of high-dimensional data and is widely used in binary classification machine learning studies (Huang et al., 2016; Nie et al., 2019; Ji et al., 2020; Zhao L. et al., 2020), including neuropsychiatric disease classification (Tang et al., 2017; Zhao et al., 2018; Zhang Y. et al., 2019; Zheng et al., 2019). The LASSO can reduce data dimensionality by compressing the unimportant feature coefficients to zero, and then a formula is generated using a linear combination of selected features that are weighted by their respective LASSO coefficients. The radiomic signature score (Rag-score) of each subject was calculated by the formula generated by the LASSO logistic regression algorithm. Rag-score is a comprehensive measurement that is calculated based on multiple significance features, and it can reflect the heterogeneity of the lesion, ROI, and volume of interest. It has been applied in radiomics-related studies and in the construction of classification models extensively (Tang et al., 2019; Ji et al., 2020; Zhao L. et al., 2020). In the 10-fold cross-validation, the primary set was divided into 10 parts: one part was left out for testing the model, and the model was trained on the remaining parts, with each part then repeated in turn. Receiver operating characteristic (ROC) curve analysis based on the Rag-score was performed, and we calculated the area under the curve (AUC), sensitivity, specificity, and accuracy to evaluate the diagnostic efficacy of the model. In the validation set, we calculated the Rag-score of each participant with the same features and the formula derived from the LASSO logistic regression algorithm in the primary set. The diagnostic performance of the model in the validation set was evaluated and validated. The threshold obtained by the ROC analysis in the primary set was used as the cutoff value to evaluate the performance. As described above, cross-validation in a small sample size leads to large error bars, and the predictive power of the fitted classifiers is arguable. We also calculated the 95% confidence interval (CI) of the AUC, sensitivity, and specificity (Gorriz et al., 2021).

### Validating the Performance

To evaluate the robustness and generalization of the model performance, we performed the LASSO logistic algorithm with 5-fold cross-validation and leave-one-out (LOO) cross-validation for feature selection. We also reported the AUC, accuracy, sensitivity, specificity, and the 95% CI to evaluate the performance of the model.

### Statistical Analysis

The statistical analysis of this study was conducted using MATLAB 2012b (MathWorks, Natick, MA, USA) and R version 3.6.1^2^. The χ_2_ test was used to assess the sex differences between patients with PD and the HCs, and the independent samples *t*-test was used to estimate the differences between PD patients and HCs in age, years of education, and MMSE scores. LASSO and ROC analyses used the “glmnt” and “pROC” software packages in R software. *P* < 0.05 was considered statistically significant.

## Results

### Clinical and Demographic Characteristics

In the primary and validation sets, there was no statistically significant difference between the HCs and PD patients in terms of sex, age, years of education, and MMSE scores (Table 1).

**Table 1:** Comparison of the general clinical data between healthy controls (HCs) and Parkinson’s disease (PD) patients in the primary and validation sets.

	Primary set		*P*-value	Validation set		*P*-value
	HCs	PD		HCs	PD	
Sex (M/F)	16/16	28/20	0.46	4/5	7/4	0.68
Age (years)	57.25 ± 4.87	55.94 ± 9.42	0.42	53.22 ± 4.41	58.73 ± 7.89	0.08
Years of education	11.12 ± 4.53	11.38 ± 3.61	0.79	11.89 ± 4.96	11.00 ± 2.61	0.61
MMSE	28.91 ± 1.73	28.52 ± 1.35	0.27	29.67 ± 0.71	29.18 ± 1.08	0.26

### Radiomic Feature Selection, Radiomic Signature Score, and Discriminative Features

**Figure 2:**
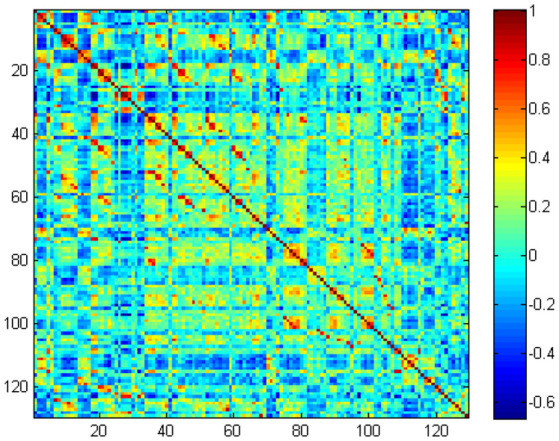
The remaining 129 features correlation heatmap.

**Figure 3:**
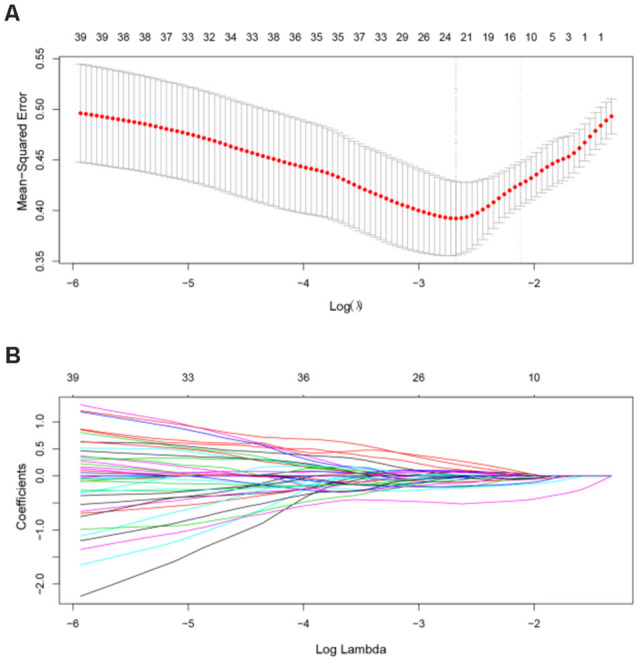
Feature selection using the LASSO model. **(A)** In the primary dataset, the penalization parameter λ was selected using 10-fold cross-validation LASSO method with the mean squared error (MSE) as the criterion. In this study, the minimum MSE was at *λ* = 0.069, log(λ) = –2.68. **(B)** LASSO coefficient profile of 129 radiomic features. There are 23 nonzero coefficient features at the optimal λ. LASSO, least absolute shrinkage and selection operator.

**Figure 4:**
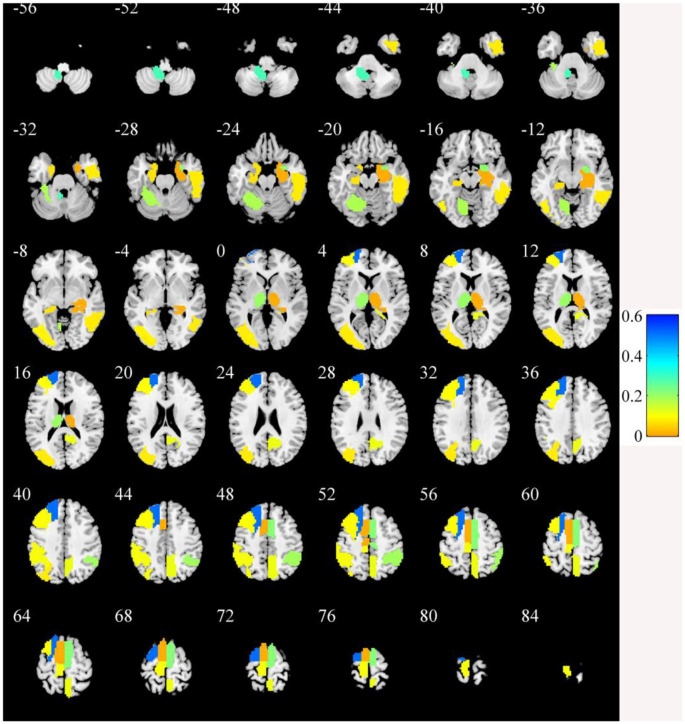
The brain regions of the selected features. The color bar value represents the feature weight value.

In the primary set, the two-way *t*-test after *Z*-standardization showed that 236 of the 1,276 features had intergroup differences (*P* < 0.05); the multicollinearity variables (correlation coefficient, *r* > 0.9) were deleted for the remaining 236 features. Finally, the remaining 129 features (Figure 2) underwent LASSO regression based on 10-fold cross-validation for further feature selection using MSE and the minimum *λ* as the feature selection criteria. When the MSE was minimum (*λ* = 0.069), there were 23 nonzero features (Figure 3), including the left middle frontal gyrus (Frontal_MidL)_mean_, right hippocampus (Hippocampus_R)_mean_, left inferior parietal gyrus (Parietal_InfL)_mean_, left paracentral lobule (Paracentral_LobuleL)_mean_, left thalamus (Thalamus_L)_mean_, left inferior cerebellum_9 (Cerebelum9L)_minimum_, left inferior occipital gyrus (Occipital_InfL)_maximum_, right inferior temporal gyrus (Temporal_InfR)_maximum_, left superior cerebellum_6 (Cerebelum6L)_maximum_, right supplementary motor area (Supp_MotorAreaR)_standard deviation_, left inferior occipital gyrus (Occipital_InfL)_standard deviation_, right precuneus (Precuneus_R)_median_, left parahippocampal gyrus (ParaHippocampal_L)_skewness_, right parahippocampal gyrus (ParaHippocampal_R)_skewness_, right amygdala (Amygdala_R)_skewness_, left middle occipital gyrus (Occipital_MidL)_skewness_, right inferior parietal gyrus (Parietal_InfR)_skewness_, left frontal middle gyrus (Frontal_MidL)_kurtosis_, left superior frontal gyrus (Frontal_SupL)_10th percentile_, left supplementary motor area (Supp_MotorAreaL)_10th percentile_, left inferior parietal gyrus (Parietal_InfL)_10th percentile_, right inferior parietal gyrus (Parietal_InfR)_10th percentile_, and the right thalamus (Thalamus_R)_10th percentile_ (Figure 4 and Table 2). The Rag-score calculation formula is as follows:

$$\begin{array}{c} Ragscore\\ \;=0.534+0.070\times Frontal\_MidL_{mean}-0.016\\ \;\times Hippocampus\_R_{mean}+0.099\times Parietal\_InfL_{mean}\\ \;-0.115\times Paracentral\_LobuleL_{mean}-0.205\\ \;\times Thalamus\_L_{mean}+0.287\times Cerebelum\_9L_{\min imum}\\ \;-0.081\times Occipital\_InfL_{\max imum}-0.081\\ \;\times Temporal\_InfR_{\max imum}-0.191\\ \;\times Cerebelum\_6L_{\max imum}-0.229\;\\ \;\times Supp\_MotorAreaR_{s\tan dard\;deviation}-0.012\;\\ \;\times Occipital\_InfL_{s\tan dard\;deviation}+0.119\\ \;\times \mathrm{Pr}ecuneus\_R_{median}+0.072\\ \;\times ParaHippocampal\_L_{skewness}+0.020\\ \;\times Parietal\_InfR_{skewness}+0.215\\ \;\times Amygdala\_R_{skewness}+0.086\;\\ \;\times Occipital\_MidL_{skewness}-0.137\\ \;\times Parietal\_InfR_{skewness}-0.037\\ \;\times Frontal\_MidL_{kurtosis}-0.513\\ \;\times Frontal\_SupL_{10th\;percentile}+0.018\\ \;\times Supp\_MotorAreaL_{10th\;percentile}+0.018\\ \;\times Parietal\_InfL_{10th\;percentile}+0.064\\ \;\times Partial\_InfR_{10th\;percentile}-0.017\\ \;\times Thalamus\_R_{10th\;percentile}. \end{array}$$

**Table 2:** Different brain regions between Parkinson’s disease patients and healthy controls.

AAL number	AAL brain areas	Brodmann brain areas	Features	Weight value
7	Frontal_MidL	BA46_L	Mean	0.070
38	Hippocampus_R	BA20_R	Mean	0.016
61	Parietal_InfL	BA40_L	Mean	0.099
69	Paracentral_LobuleL	BA4_L	Mean	0.115
77	Thalamus_L	None	Mean	0.205
105	Cerebelum_9L	None	Minimum	0.287
53	Occipital_InfL	BA19_L	Maximum	0.081
90	Temporal_InfR	BA20_R	Maximum	0.081
99	Cerebelum_6L	BA19_L	Maximum	0.191
20	Supp_Motor_AreaR	BA6_R	Standard deviation	0.229
53	Occipital_InfL	BA19_L	Standard deviation	0.012
68	Precuneus_R	None	Median	0.119
39	ParaHippocampal_L	BA35_L	Skewness	0.072
40	ParaHippocampal_R	BA35_R	Skewness	0.020
42	Amygdala_R	BA34_R	Skewness	0.215
51	Occipital_MidL	BA19_L	Skewness	0.086
62	Parietal_InfR	BA40_R	Skewness	0.137
7	Frontal_MidL	BA46_L	Kurtosis	0.037
3	Frontal_SupL	None	10th percentile	0.513
19	Supp_Motor_AreaL	BA6_L	10th percentile	0.016
61	Parietal_InfL	BA40_L	10th percentile	0.018
62	Parietal_InfR	BA40_R	10th percentile	0.064
78	Thalamus_R	None	10th percentile	0.017

*AAL, Automated Anatomical Labeling; BA, Brodmann brain areas; Frontal_Mid, middle frontal gyrus; *Parietal_Inf*, inferior parietal gyrus; Occipital_Inf, inferior occipital gyrus; Temporal_Inf, inferior temporal gyrus; *Supp_MotorArea*, supplementary motor area; *Occipital_Inf*, inferior occipital gyrus; *Occipital_Mid*, middle occipital gyrus; *Frontal_Mid*, frontal middle gyrus; *Frontal_Sup*, superior frontal gyrus; *L*, left; *R*, right*.

**Figure 5:**
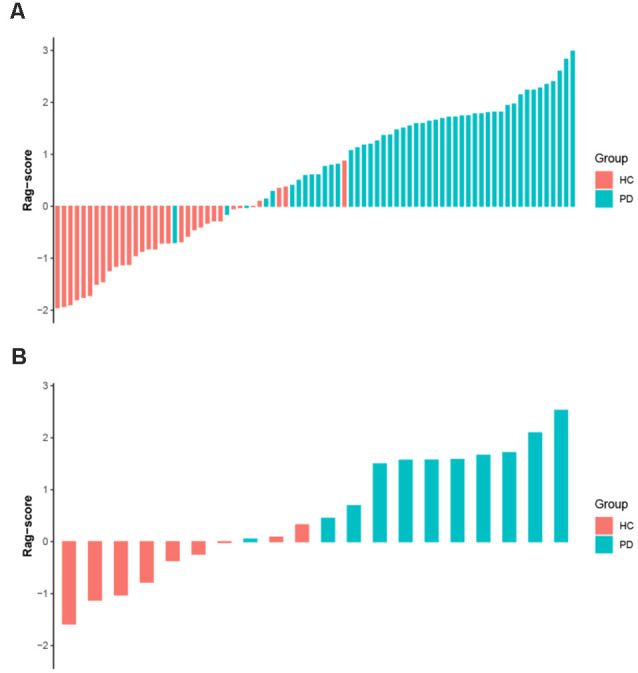
The Rag-score for each subject in the primary **(A)** and validation **(B)** sets. Red bars represent the HC group and blue bars represent the PD group. The greater the score, the more likely it is to be PD. *Rag-scores*, radiomic signature scores; *HC*, healthy control; *PD*, Parkinson’s disease.

The Rag-scores of each subject are shown in Figure 5. It can be seen from the figure that the Rag-score can distinguish HCs and PD patients well in both the primary and validation sets.

### Model Evaluation

**Figure 6:**
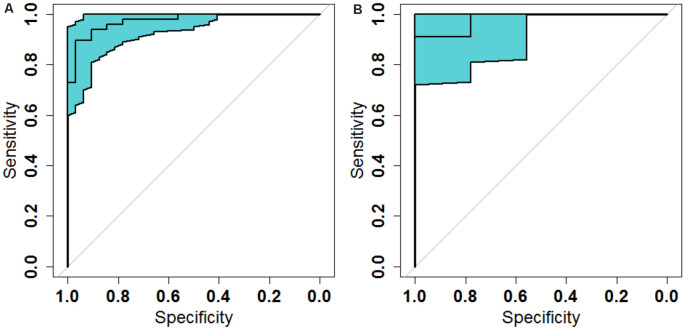
Receiver operating characteristic (ROC) analysis of the radiomic signature scores in the primary **(A)** and validation **(B)** sets.

The ROC analysis of the Rag-scores in the primary set showed that the AUC was 0.974. When the cutoff value was 0.117, the sensitivity was 93.8%, the specificity was 90.6%, and the accuracy was 93.8% (Figure 6A and Table 3). The accuracy of the validation set was 90.0%, with a primary set threshold of 0.117 as the standard, and the sensitivity and specificity of the validation set were 90.9% and 88.9%, respectively (Figure 6B and Table 3).

**Table 3:** Classification performances of the different cross-validation methods.

CV methods	Dataset	AUC (95% CI)	Accuracy (%)	Sensitivity (%; 95% CI)	Specificity (%; 95% CI)
10-fold	Primary	0.974 (0.946–1)	93.8	93.8 (82.8–98.69)	90.6 (74.98–98.02)
	Validation	0.980 (0.929–1)	90.0	90.9 (58.72–99.77)	88.9 (51.75–99.72)
5-fold	Primary	0.978 (0.953–1)	90.0	97.9 (88.93–99.95)	78.1 (60.03–90.72)
	Validation	0.981 (0.925–1)	90.0		77.8 (39.99–97.19)
LOO	Primary	0.971 (0.940–1)	83.8	75.0 (60.4–86.36)	96.9 (83.78–99.92)
	Validation	0.968 (0.918–1)	85.0	72.7 (39.03–93.98)	

*CV, cross-validation; *LOO*, leave one out; *AUC*, area under the curve; *CI*, confidence interval*.

### Validation Analysis

To assess the effect of the cross-validation method, we repeated the LASSO regression process with 5-fold and LOO cross-validations. We found that our models achieved high classification performances with the 5-fold cross-validation and LOO cross-validation in both the primary and validation sets. However, the CIs were relatively wide, especially in the validation set (Table 3).

## Discussion

In this study, we used the ALFF measurement of rs-fMRI data to perform feature selection and data dimensionality reduction by two-way independent samples *t*-test and LASSO logistic regression based on 10-fold cross-validation. We calculated the participants’ Rag-score and performed ROC analysis based on the selected features. The classification model had a high diagnostic efficiency, with an AUC of 0.966, a sensitivity of 92.9%, a specificity of 97.6%, and an accuracy of 95.2%. These results demonstrate that histogram analysis based on rs-fMRI can be used for the diagnosis of PD.

Radiomics was first proposed by Lambin et al. (2012). It can extract high-throughput quantitative features of medical images and can provide greater features than conventional image analysis methods, thus improving disease diagnosis and prognostic evaluation (Nie et al., 2019; Tang et al., 2019; Ji et al., 2020). It was first used for tumor heterogeneity evaluation, tumor diagnosis and differential diagnosis (Nie et al., 2019), prognostic evaluation (Zhao L. et al., 2020), and tumor recurrence (Tang et al., 2019; Ji et al., 2020), among other uses. Recent studies have also indicated that it has a great application value in neuropsychological diseases (Tang et al., 2017; Péran et al., 2018; Sun et al., 2018; Huang K. et al., 2019). Different from the previous radiomic studies in the field of oncology, they often have only one ROI. However, in our study, there are 116 brain ROIs. We therefore only extracted the first-order intensity statistical features—histogram features; otherwise, our model will have too many features. It is easy for a model to fall into a “curse of dimension” and model overfitting.

Rs-fMRI is widely used in the study of PD (Gu et al., 2016; Amoroso et al., 2018; Hohenfeld et al., 2018; Rispoli et al., 2018), and ALFF is one of its commonly used metrics (Hu et al., 2015; Gu et al., 2016). In this study, the ROI-based method (Tang et al., 2017; Sun et al., 2018) was used to extract the histogram features of the ALFF images in specific brain areas using the AAL 116 atlas, and the machine learning method was used for data dimensionality reduction and modeling to explore the diagnostic value of the rs-fMRI-based machine learning in PD.

In this study, we standardized the data before data analysis. This procedure is widely used in the preprocessing of machine learning, which can effectively reduce the influence of the different units between features or reduce the influence of signal changes in a larger image signal range, which is beneficial to the improvement of model performance (Pereira et al., 2009; Tang et al., 2019). Among them, the *Z*-standardization (Tang et al., 2019; Wang et al., 2020) used in this research is one of the most commonly used methods.

Most previous studies have extracted features based on the brain atlas, which only extracted the mean values of the metrics (such as ALFF, fractional anisotropy, mean diffusivity, regional homogeneity, functional connectivity, voxel-mirrored homotopic connectivity, etc.) in the ROI defined by the brain atlas (Dai et al., 2012; Cui et al., 2016; Ding et al., 2017; Tang et al., 2017; Sun et al., 2018; Zhou et al., 2020). In our study, we extracted not only the mean ALFF values in the predefined ROIs but also other histogram features, including the minimum, maximum, range, standard deviation, variance, median, skewness, kurtosis, 10th percentile, and the 90th percentile, which could more comprehensively reflect ALFF information in the predefined ROIs. Our method achieved great classification performance on both the primary set and the validation set. We found that these features could be used to distinguish PD patients and HCs, which indicated that these features have physiological significance. Tang et al. (2017) combined the mean diffusion tensor imaging metrics to detect HIV patients. The accuracy and AUC of the model were 83.08% and 0.911, respectively. A machine learning study using ALFF metrics to detect PD used the AAL 116 atlas to extract the mean ALFF features. The accuracy and AUC were 80.75% and 0.8109, respectively. Another study used the Harvard–Oxford atlas to extract the mean ReHo, ALFF, VMHC, gray matter volume, and FC features. The authors found that both random forest (accuracy = 0.8261, AUC = 0.9015) and support vector machine (accuracy = 0.8483, AUC = 0.9697) achieved the perfect accuracy and AUC for distinguishing between PD and HC subjects (Cao et al., 2020). Our results are better than those of these studies, which may indicate that our histogram analysis method can extract more information in the ROIs (Lambin et al., 2012; Gillies et al., 2016).

Radiomics can extract a large number of quantitative features; however, its data dimensionality is too high when compared with the sample size of most studies, making it is easy to fall into a “curse of dimensionality,” thus causing the model to overfit. Hence, the features must be selected for dimensionality reduction to obtain the most valuable features in order to improve the reliability and accuracy of the model (Gu et al., 2016; Péran et al., 2018; Rubbert et al., 2019; Wang et al., 2020). In our study, we performed a two-way independent samples *t*-test on the standardized data to select the features that were significantly different between groups for subsequent analysis. Then, we applied previous research data processing methods (Mo et al., 2019; Tang et al., 2019; Wang et al., 2020) and used correlation analysis to remove the features with high correlation coefficients in order to reduce the multicollinearity of variables. We chose a correlation coefficient threshold of 0.9 to remove highly correlated variables (Tang et al., 2019). The LASSO logistic regression is very suitable for high-dimensional data processing. It can select the most predictive radiomic features and compress the non-important feature coefficients to zero in order to achieve the purpose of data dimensionality reduction and feature selection (Huang K. et al., 2019; Wang et al., 2020). The linear combination of the selected features weighted by their respective coefficients was used to calculate the Rag-score of each subject, and the resulting Rag-score was analyzed by ROC analysis to evaluate the diagnostic efficacy of the model. In this study, LASSO based on 10-fold cross-validation was used for further dimensionality reduction and feature selection of the data; at the same time, the Rag-score of each subject was calculated and ROC analysis was performed to evaluate the performance of the model.

Previous studies (Varoquaux, 2018; Gorriz et al., 2021) have demonstrated that cross-validation in a small sample size leads to large error bars. Varoquaux (2018) also pointed out that the best way to solving the problem was to test the model performance across several datasets. Therefore, we divided the dataset into a primary set and an independent validation set, and we tested the model performance using the independent validation set. In our study, we found that our models achieved high classification performances with different cross-validation methods both in the primary and validation sets. However, the error bars were relatively large, especially in the validation set. Our findings are consistent with those of previous studies (Varoquaux, 2018; Gorriz et al., 2021). In spite of the arguable power of the model, cross-validation is the best tool available because it is the only non-parametric method to test for model generalization and it can measure the ability of the model to predict new data (Varoquaux, 2018). Currently, cross-validation is still the most popular method in use.

In this study, the discriminative brain areas that can be used for PD diagnosis are located in the frontal, temporal, parietal, occipital, and limbic lobes, as well as in the cerebellum and the thalamus, which are consistent with the results of previous PD studies (Szewczyk-Krolikowski et al., 2014; O’Callaghan et al., 2016; Chen et al., 2017; Tuovinen et al., 2018). Many studies have indicated that damage to the corticothalamus–striatum–cortical pathway and the reciprocal striatum–cerebellar loop is the basis of many clinical symptoms of PD, and the thalamus plays an important role in function regulation and conduction (Szewczyk-Krolikowski et al., 2014; O’Callaghan et al., 2016; Tuovinen et al., 2018). In this study, we found that the radiomic features of the bilateral thalamus are helpful in the diagnosis of PD. A previous study (O’Callaghan et al., 2016) showed that multiple brain areas of the cerebellum of PD patients were atrophied, including the bilateral lobules I–IV, VI, VII (crus I and crus II), VIIb, VIIIa, VIIIb, right V, and the cerebellar vermis. The study also indicated that the modulatory relationship of the subthalamic nucleus on intracerebellar connectivity was lost in PD patients and that there were extensive cerebellar–cortical network abnormalities. Tuovinen et al. (2018) found that the connections within the cerebellum and between the cerebellum and the sensorimotor network in PD patients increased, the connections between the cerebellum and the caudate nucleus, thalamus, and amygdala increased, and the connection between the supplementary motor area and the cingulate gyrus decreased. Chen et al. (2017) found that the bilateral cerebellar gray matter volume was reduced and that the functional connections among the bilateral cerebellum, angular gyrus, hippocampus, middle occipital gyrus, and posterior cingulate gyrus were abnormal. Hu et al. (2015) also showed that PD patients had cerebellum ALFF abnormalities and abnormal functional connections between the cerebellum and the left middle cingulate gyrus. In our study, we also found extensive brain dysfunction in the cerebellum and multiple gray matter areas, which is consistent with the findings of a previous study. Besides, previous studies (Szewczyk-Krolikowski et al., 2014; Hu et al., 2015; Lucas-Jiménez et al., 2015; Hou et al., 2016; Li et al., 2016; Huang L. C. et al., 2019) also found structural and functional abnormalities in the insula, lingual gyrus, paracentral lobule, right inferior temporal gyrus, parahippocampal gyrus, precuneus, middle frontal cortex, dorsolateral prefrontal lobe, and other brain areas, which were similar to the results of the present study. This study showed that radiomics based on rs-fMRI can effectively identify abnormal brain activity areas in PD patients and provide support for the interpretation of PD neural mechanisms.

This study has some limitations. Firstly, as obtaining a large dataset is difficult in neuroimaging, where data acquisition is costly, the sample size of this study is relatively limited, but compared with the sample sizes of previous intergroup comparison studies (Lei et al., 2017; Zhou M. et al., 2019) and some machine learning articles (Hou et al., 2016; Tang et al., 2017), the sample size of this study is relatively large. Previous studies (Sun et al., 2018; Huang K. et al., 2019) have shown that multimodal imaging and clinical indicators can improve the performance of machine learning models. This study only selected the ALFF of rs-fMRI as input variables. Subsequent work can incorporate more rs-fMRI metrics, different functional imaging sequences, and clinical measurements to explore its effect on the classification model. In this study, both the primary and validation sets confirmed that the model has good classification accuracy, but no external validation was performed to further verify the generalization ability of the model. Other machine learning models or methods, such as support vector machine (Zhou et al., 2020), convolution neural network (Zhang J. et al., 2019), deep neural network (Liu et al., 2020), and transfer learning (Wang S. et al., 2019), have also been applied in previous neuroimaging studies, and they can obtain great classification performance. A previous study (Wang S. H. et al., 2019) used AlexNet as the basic transfer learning model to identify alcoholism. The method yielded a sensitivity of 97.44 ± 1.15%, a specificity of 97.41 ± 1.51%, and an accuracy of 97.42 ± 0.95% on the test set. In another study (Wang S. H. et al., 2019) employing densely connected neural network as the basic algorithm for transfer learning to detect cerebral micro-bleedings, the model achieved 97.71% classification accuracy. In the future, we will try other machine learning methods to detect PD.

In conclusion, this study used rs-fMRI data to extract the histogram features of brain regions based on brain atlases and used machine learning analysis to build a PD classification model. The study showed that this method can classify PD patients and HCs well and effectively identify PD patients’ regions of abnormal brain function; furthermore, it can assist in the early diagnosis of PD and provide a means for PD mechanism research and clinical therapeutic efficacy evaluation.

## Data Availability Statement

The original contributions presented in the study are included in the article, further inquiries can be directed to the corresponding author.

## Ethics Statement

The studies involving human participants were reviewed and approved by Medical Research Ethical Committee of Nanjing Brain Hospital. The ethics committee waived the requirement of written informed consent for participation.

## Author Contributions

DS conducted the experiment, performed the data processing and analysis, and wrote the manuscript. HZ, GW, and SW collected the data and performed the data processing and analysis. KR supervised the whole study, including experiments and manuscript writing. All authors contributed to the article and approved the submitted version.

## Conflict of Interest

The authors declare that the research was conducted in the absence of any commercial or financial relationships that could be construed as a potential conflict of interest.

## Footnotes

^1^http://dx.doi.org/10.6084/m9.figshare.1433996

^2^https://cran.r-project.org/
